# Systematic detection of brain protein-coding genes under positive selection during primate evolution and their roles in cognition

**DOI:** 10.1101/gr.262113.120

**Published:** 2021-03

**Authors:** Guillaume Dumas, Simon Malesys, Thomas Bourgeron

**Affiliations:** 1 Human Genetics and Cognitive Functions, Institut Pasteur, UMR3571 CNRS, Université de Paris, Paris 75015, France;; 2 Department of Psychiatry, Université de Montreal, CHU Sainte-Justine Hospital, Montreal H3T 1C5, Quebec, Canada

## Abstract

The human brain differs from that of other primates, but the genetic basis of these differences remains unclear. We investigated the evolutionary pressures acting on almost all human protein-coding genes (*N* = 11,667; 1:1 orthologs in primates) based on their divergence from those of early hominins, such as Neanderthals, and non-human primates. We confirm that genes encoding brain-related proteins are among the most strongly conserved protein-coding genes in the human genome. Combining our evolutionary pressure metrics for the protein-coding genome with recent data sets, we found that this conservation applied to genes functionally associated with the synapse and expressed in brain structures such as the prefrontal cortex and the cerebellum. Conversely, several genes presenting signatures commonly associated with positive selection appear as causing brain diseases or conditions, such as micro/macrocephaly, Joubert syndrome, dyslexia, and autism. Among those, a number of DNA damage response genes associated with microcephaly in humans such as *BRCA1*, *NHEJ1*, *TOP3A*, and *RNF168* show strong signs of positive selection and might have played a role in human brain size expansion during primate evolution. We also showed that cerebellum granule neurons express a set of genes also presenting signatures of positive selection and that may have contributed to the emergence of fine motor skills and social cognition in humans. This resource is available online and can be used to estimate evolutionary constraints acting on a set of genes and to explore their relative contributions to human traits.

Modern humans (*Homo sapiens*) can perform complex cognitive tasks well and communicate with their peers (Dunbar and Shultz 2017). Anatomic differences between the brains of humans and other primates are well documented (e.g., cortex size, prefrontal white matter thickness, lateralization), but how the human brain evolved remains a matter of debate (Varki et al. 2008). A recent study of endocranial casts of *Homo sapiens* fossils indicates that brain size in early *Homo sapiens*, 300,000 yr ago, was already within the range of that in present-day humans (Neubauer et al. 2018). However, brain shape evolved more gradually within the *Homo sapiens* lineage, reaching its current form between about 100,000 and 35,000 yr ago. It has also been suggested that the enlargement of the prefrontal cortex relative to the motor cortex in humans is mirrored in the cerebellum by an enlargement of the regions of the cerebellum connected to the prefrontal cortex (Balsters et al. 2010). These anatomic processes of tandem evolution in the brain paralleled the emergence of motor and cognitive abilities, such as bipedalism, planning, language, and social awareness, which are mainly well developed in humans.

Genetic differences in primates undoubtedly contributed to these brain and cognitive differences, but the genes or variants involved remain largely unknown. Indeed, demonstrating that a genetic variant is adaptive requires strong evidence at both the genetic and functional levels. Only a few genes have been shown to be human-specific. They include *SRGAP2C* (Charrier et al. 2012), *ARHGAP11B* (Florio et al. 2015), and *NOTCH2NLA* (Suzuki et al. 2018), which emerged through recent gene duplication in the *Homo* lineage (Dennis et al. 2017). The expression of these human-specific genes in the mouse brain expands cortical neurogenesis (Dennis et al. 2012; Florio et al. 2015; Nuttle et al. 2016; Suzuki et al. 2018). Several genes involved in brain function display accelerated coding region evolution in humans. For example, *FOXP2* has been associated with verbal apraxia and *ASPM* with microcephaly (Enard et al. 2002; Montgomery et al. 2014). Functional studies have also shown that mice carrying a “humanized” version of *FOXP2* display qualitative changes in ultrasonic vocalization (Enard et al. 2009). However, these reports targeting only specific genes sometimes provide contradictory results (Atkinson et al. 2018). Other studies have reported sequence conservation to be stronger in the protein-coding genes of the brain than in those of other tissues (Miyata et al. 1994; Wang et al. 2006; Tuller et al. 2008), suggesting that the primary substrate of evolution in the brain is regulatory changes in gene expression (King and Wilson 1975; Pollard et al. 2006; Changeux 2017) and splicing (Calarco et al. 2007). In addition, several recent studies have explored the genes subjected to the highest degrees of constraint during primate evolution or in human populations to improve estimations of the pathogenicity of variants identified in patients with genetic disorders (Sundaram et al. 2018; Havrilla et al. 2019). In contrast, fewer studies have systematically detected genes that diverged during primate evolution (Dorus et al. 2004; Nielsen et al. 2005; Huang et al. 2013).

We describe here an exhaustive screening of all protein-coding genes for conservation and divergence from the common primate ancestor, making use of rich data sets of brain single-cell transcriptomics, proteomics, and imaging to investigate the relationships between these genes and brain structure, function, and diseases.

## Results

### Strong conservation of brain protein-coding genes

**Figure 1.**
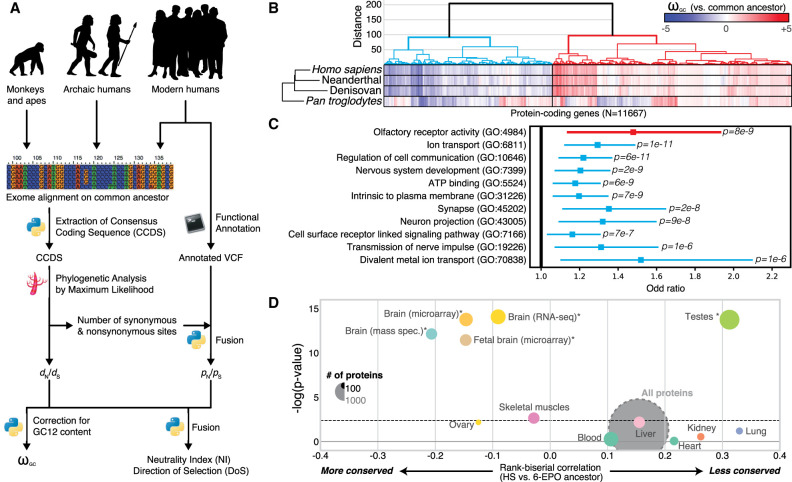
Evolution of protein-coding genes across tissues and biological functions. (*A*) Analysis pipeline for the extraction of ω_GC12_, a corrected and normalized measurement of the evolution of protein-coding genes that behaves like a *Z*-score and takes into account the GC content of codons. (*B*) Hierarchical clustering, based on ω_GC12_, across all protein-coding genes (1:1 orthologs in hominins with medium coverage) (Supplemental Table S1). (*C*) Gene Ontology (GO) enrichments for the red and blue clusters in *B* (for all GO terms, see Supplemental Table S2). Horizontal lines indicate 95% confidence intervals. (*D*) Funnel plot summarizing the evolution of protein-coding genes specifically expressed in different tissues of the human body (Supplemental Table S3). Horizontal and vertical axes indicate, respectively, the effect size and the statistical significance. Circle size indicates the number of proteins in the set. The dashed horizontal line indicates the threshold for significance after Bonferroni correction. Stars indicate the set of genes for which statistical significance was achieved in multiple comparisons after correction, with a bootstrap taking GC12 content and coding sequence length into account. (HS) *Homo sapiens*; (6-EPO ancestor) the reconstructed ancestral genome of primates based on alignments of *Homo sapiens*, chimpanzee, gorilla, orangutan, rhesus macaque, and marmoset genomes.

We first compared the sequences of modern humans, archaic humans, and other primates to those of their common primate ancestor (inferred from the Compara six-way primate Enredo, Pecan, Ortheus multiple alignments) (Paten et al. 2008) to extract a measurement of evolution for 11,667 of the 1:1 orthologs across primates, selected from the 17,808 protein-coding genes in the modern human genome (available online from https://genevo.pasteur.fr/) (Fig. 1A; see also Supplemental Figs. S1, S2; Kapheim et al. 2015). Our measurement is derived from one of the most widely used and reliable measurements of evolutionary pressure on protein-coding regions, the *d*_N_/*d*_S_ ratio (Yang and Bielawski 2000), also called ω. This measurement compares the rates of nonsynonymous and synonymous mutations of coding sequences. More nonsynonymous mutations than expected signifies positive selection, and fewer signifies selective constraint. We first estimated *d*_N_ and *d*_S_ for all 1:1 orthologous genes, because the evolutionary constraints on duplicated genes are relaxed (only the Y Chromosome was excluded from these analyses) (O'Toole et al. 2018). We then adjusted the *d*_N_/*d*_S_ ratio for biases induced by variations of mutation rate with the GC content of codons. Finally, we renormalized the values obtained for each taxon across the whole genome. The final ω_GC12_ obtained took the form of *Z*-score corrected for GC content that quantified the unbiased divergence of genes relative to the ancestral primate genome (Kapheim et al. 2015). High positive ω_GC12_ indicates a genetic signature commonly, but not exclusively, associated with positive evolutionary selection; on the contrary, negative ω_GC12_ reflects selective constraint.

Using the ω_GC12_ for all protein-coding genes in *Homo sapiens*, Denisovans, Neanderthals, and *Pan troglodytes*, we identified two distinct clusters in hominins (Fig. 1B; Supplemental Table S1): one containing “positively selected” genes (PSGs), enriched in olfactory genes (OR = 1.48, *P* = 8.4 × 10^−9^), and one with genes under “selective constraint” (SCGs), enriched in brain-related biological functions (Fig. 1C; Supplemental Table S2). This second cluster revealed particularly strong conservation of genes encoding proteins involved in nervous system development (OR = 1.2, *P* = 2.4 × 10^−9^) and synaptic transmission (OR = 1.35, *P* = 1.7 × 10^−8^).

We investigated the possible enrichment of specific tissues in PSGs and SCGs by analyzing RNA-seq (Illumina Bodymap2 and GTEx), microarray, and proteomics data sets (Methods). For expression data, despite that virtually no gene is expressed only in one tissue, we calculated a tissue specificity score for each gene by normalizing their profile across tissues (for more details, see Supplemental Fig. S3). The results confirmed a higher degree of conservation for protein-coding genes more specifically expressed in the brain (Wilcoxon rank correlation rc = −0.1, *P* = 4.1 × 10^−12^, bootstrap-corrected for gene length and GC content) than for those expressed elsewhere in the body, with the greatest divergence observed for genes expressed in the testis (Wilcoxon rc = 0.3, *P* = 7.8 × 10^−11^, bootstrap-corrected for gene length and GC content) (Fig. 1D; for a replication with GTEx data, see also Supplemental Table S3; Supplemental Fig. S4). This conservation of brain protein-coding genes was replicated with two other data sets (MicroArray: Wilcoxon OR = −0.18, *P* = 1.8 × 10^−12^; mass spectrometry: Wilcoxon rc = −0.21, *P* = 1.55 × 10^−9^; bootstrap-corrected for gene length and GC content).

### Conservation of protein-coding genes relating to nervous system substructure and neuronal functions

**Figure 2.**
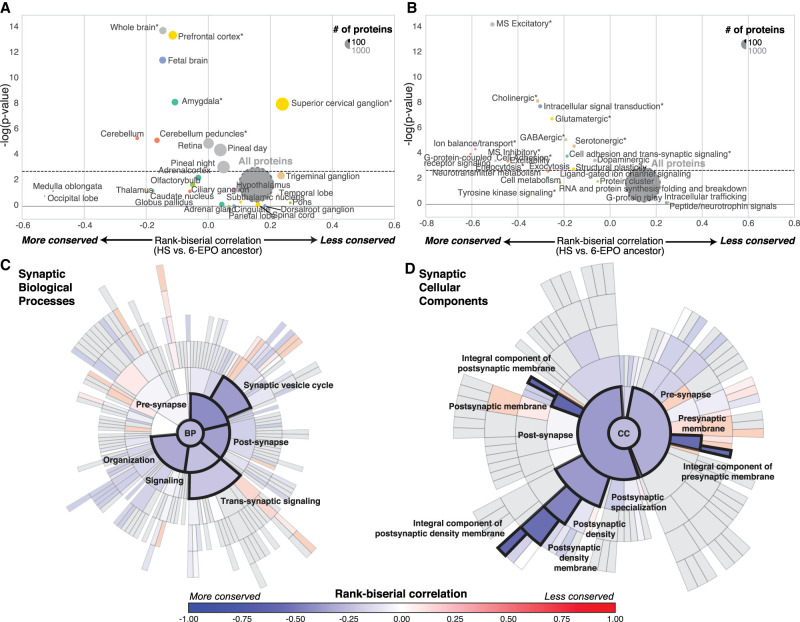
Evolution of brain-related protein-coding genes. (*A*,*B*) Funnel plots summarizing the evolution of protein-coding genes specifically expressed in brain substructures (*A*) and synaptic functions (*B*); the dashed horizontal line indicates the threshold for significance after Bonferroni correction. Stars indicate sets of genes for which statistical significance was achieved for multiple comparisons with bootstrap correction. (*C*,*D*) SynGO sunburst plots showing nested statistically conserved (blue) biological processes (*C*) and cellular components (*D*) of the synapse. The circle in the *center* represents the root node, with the hierarchy moving outward from the *center*. A segment of the *inner* circle bears a hierarchical relationship to those segments of the *outer* circle which lie within the angular sweep of the parent segment.

We then used microarray (Su et al. 2004) and RNA-seq (The GTEx Consortium 2015) data to investigate the evolutionary pressures acting on different regions of the central nervous system. Three central nervous system substructures appeared to have evolved under the highest level of purifying selection at the protein sequence level (ω_GC12_ < 2): (1) the cerebellum (Wilcoxon rc = −0.29, *P* = 5.5 × 10^−6^, Bonferroni-corrected) and the cerebellar peduncle (Wilcoxon rc = −0.11, *P* = 3.2 × 10^−4^, bootstrap-corrected for gene length and GC content); (2) the amygdala (Wilcoxon rc = −0.11, *P* = 4.1 × 10^−6^, bootstrap-corrected for gene length and GC content); and (3) the prefrontal cortex (Wilcoxon rc = −0.1, *P* = 5.7 × 10^−10^, bootstrap-corrected for gene length and GC content) (Fig. 2A; Supplemental Table S3). Indeed, it has been suggested that the prefrontal cortex is one of the most divergent brain structures in human evolution (Schoenemann et al. 2005), this diversity being associated with high-level cognitive function (Frith and Dolan 1996). Only one brain structure was expressing more PSG than expected: the superior cervical ganglion (Wilcoxon rc = 0.22, *P* = 1 × 10^−6^, bootstrap-corrected for gene length and GC content). This structure provides sympathetic innervation to many organs and is associated with the archaic functions of the fight-or-flight response. The PSGs expressed in the superior cervical ganglion include *CARF*, which was found to be specifically divergent in the genus *Homo*. This gene encodes a calcium-responsive transcription factor that regulates the neuronal activity-dependent expression of *BDNF* (Tao et al. 2002) and a set of singing-induced genes in the song nuclei of the zebra finch, a songbird capable of vocal learning (Whitney et al. 2014). This gene had a raw *d*_N_/*d*_S_ of 2.44 (seven nonsynonymous vs. one synonymous mutation in *Homo sapiens* compared to the common primate ancestor) and was found to be one of the PSGs with the higher *d*_N_/*d*_S_ value expressed in the human brain.

We then investigated the possible enrichment of PSGs and SCGs in brain-specific Gene Ontology terms. All pathways displayed high overall levels of conservation, but genes encoding proteins involved in glutamatergic and GABAergic neurotransmission were generally more conserved (Wilcoxon rc = −0.25; *P* = 9.8 × 10^−6^, Bonferroni-corrected) than those encoding proteins involved in dopamine and peptide neurotransmission and intracellular trafficking (Fig. 2B; Supplemental Table S3). The recently released ontology of the synapse provided by the SynGO consortium (http://syngoportal.org) was incorporated into this analysis, not only confirming the globally strong conservation of the synapse but also revealing its close relationship to trans-synaptic signaling processes (Wilcoxon rc = −0.21, *P* = 4.5 × 10^−5^, Bonferroni-corrected) and to postsynaptic (rc = −0.56, *P* = 6.3 × 10^−8^, Bonferroni-corrected) and presynaptic membranes (Wilcoxon rc = −0.56, *P* = 7 × 10^−8^, Bonferroni-corrected) (Fig. 2C,D).

### Positively selected genes and their correlation with brain expression and function

**Figure 3.**
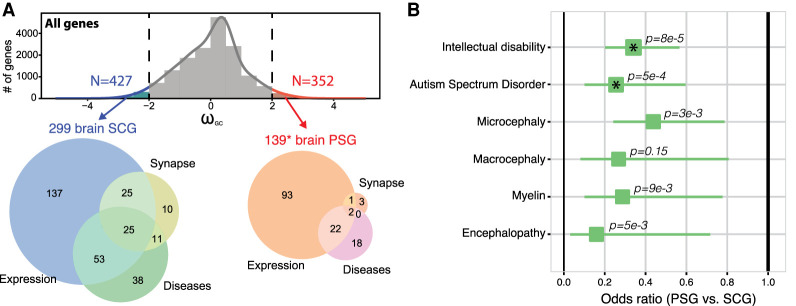
Brain protein-coding genes and human diseases. (*A*) Distribution of ω_GC12_ and Venn diagrams describing SCGs and PSGs situated at the extremes of the ω_GC12_ distribution (>2SD) specifically expressed in the brain (genes with specificity *Z*-score > 2 in any brain-related tissues of Figs. 1D and 2A), related to the synapse or brain diseases (Supplemental Table S4). (*) Addition of four genes (*FARSB*, *KRT14*, *NPHS1*, *RSPH1*) containing *Homo sapiens*–specific mutations predicted as deleterious (CADD>15). (*B*) Odds ratios for protein-coding gene sets related to brain diseases (Fisher's exact test; asterisks indicate *P*-values significant after Bonferroni correction; horizontal lines indicate 95% confidence intervals).

We focused on the genes situated at the extremes of the ω_GC12_ distribution (>2SD) (Fig. 3A; Supplemental Table S4) and those fixed in the modern *Homo sapiens* population (neutrality index < 1), to ensure that we analyzed PSGs with signs of strong positive selection. Only 139 of these 352 highly PSGs were brain-related (impoverishment for brain genes, Fisher's exact test OR = 0.66, *P* = 1 × 10^−4^), listed as synaptic genes (Ruano et al. 2010; Lips et al. 2012), specifically expressed in the brain (+2SD for specific expression) or related to a brain disease (extracted systematically from Online Mendelian Inheritance in Man [OMIM]: https://www.omim.org; and Human Phenotype Ontology [HPO]: https://hpo.jax.org/app/). For comparison, we also extracted the 427 SCGs under very strong selective constraint, 299 of which were related to the brain categories listed above (enrichment for brain genes, Fisher's exact test OR = 1.26, *P* = 0.0032).

**Figure 4.**
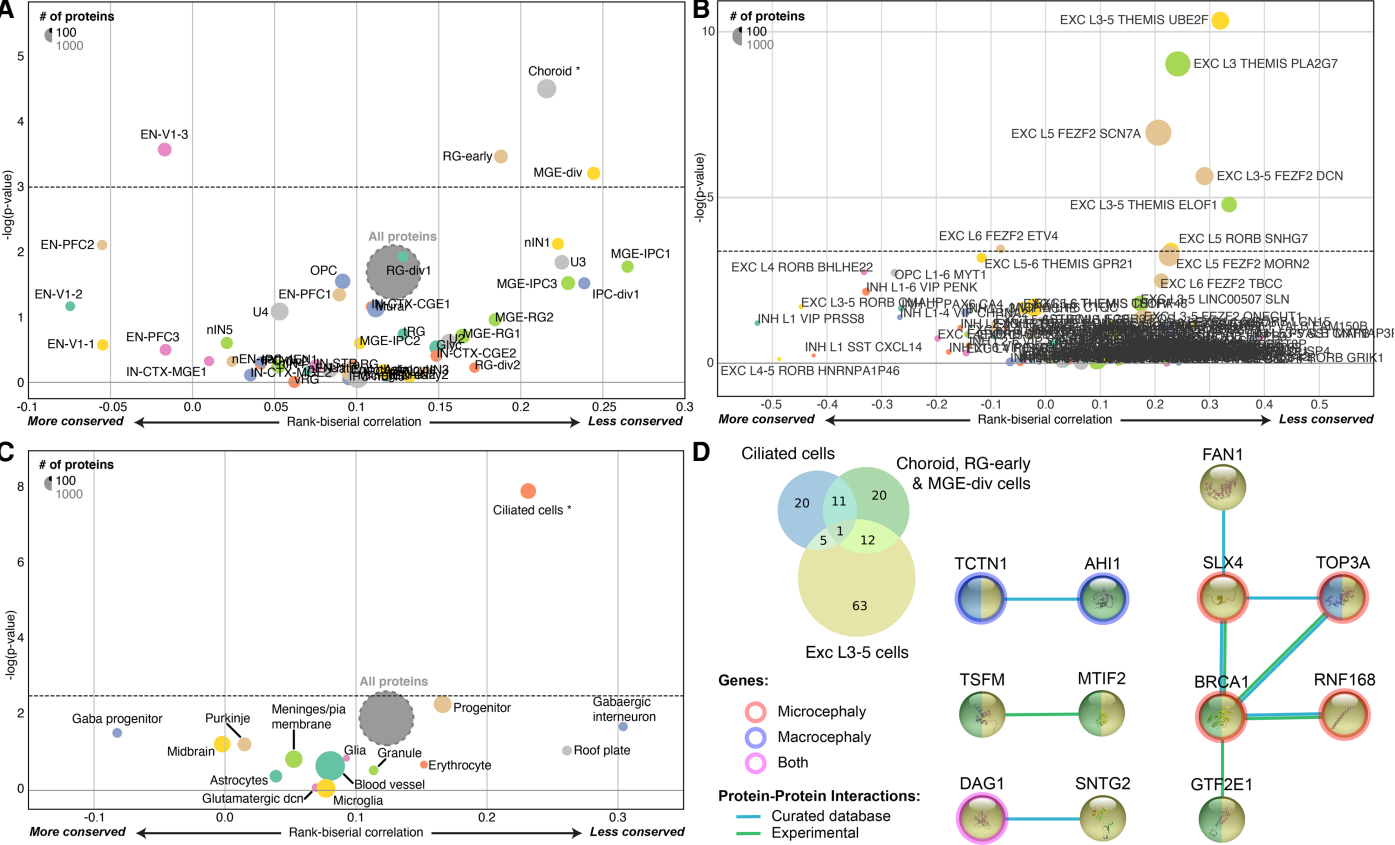
Evolution of protein-coding genes expressed in different cell types. (*A*–*C*) Funnel plots summarizing the evolution of protein-coding genes specifically expressed in different cell types within the human cerebral cortex (Supplemental Table S6; Nowakowski et al. 2017) (*A*), human cortical layers (Supplemental Table S6; Hodge et al. 2019; Tasic et al. 2018) (*B*), and the mouse cerebellum (Supplemental Table S6; Carter et al. 2018) (*C*). (*D*) Venn diagram of the PSGs expressed specifically in those cell types, with the corresponding Protein–Protein Interaction network (STRINGdb) (Jensen et al. 2009) and their annotated association with micro- and macrocephaly (HPO) (Köhler et al. 2019). (EN-V1) primary visual cortex neurons; (RG-early) radial glia early cortical progenitors; (MGE-div) medial ganglionic eminence dividing cells; (Exc) excitatory; (L3-5) layers 3–5; THEMIS, UBE2F, PLA2G7, and so forth are cell type markers.

Using these 427 SCGs and 352 PSGs, we first used the Brainspan data available from the specific expression analysis (SEA) to confirm that the population of genes expressed in the cerebellum and the cortex was enriched in SCGs (Supplemental Fig. S5). Despite this conservation, based on the adult Allen Brain Atlas, we identified a cluster of brain subregions (within the hypothalamus, cerebral nuclei, and cerebellum), more specifically expressing PSGs (Supplemental Fig. S6). Analyses of the human cerebral cortex single-cell RNA-seq (Fig. 4A; Supplemental Table S5; Nowakowski et al. 2017) also revealed an excess of PSGs expressed in the choroid plexus (the primary function is to produce cerebrospinal fluid), in the medial ganglionic eminence (MGE-div; implicated in the production of GABAergic interneurons and their migration to the neocortex during development) (Brazel et al. 2003), and the radial glial (RG) cells.

Using a second RNA-seq data set from the human cortex (Tasic et al. 2018; Hodge et al. 2019), we identified five cell types, all from layer 3 or 5, expressing PSGs more than expected using a stringent Bonferroni and bootstrap correction for gene length and GC content (Fig. 4B; Supplemental Table S5). Among them, two groups of excitatory neurons, THEMIS-PLA2G7 and FEZF2-SCN7A, express several PSGs involved in DNA damage response (Arcas et al. 2014) and mutated in patients with microcephaly such as *BRCA1*, *NHEJ1*, *RNF168*, and *TOP3A*.

We investigated organoid and human cortex data sets that previously revealed seven clusters of cells (Camp et al. 2015). Overall, the marker genes of these clusters are on average strongly constrained compared to the rest of the genome (Supplemental Fig. S7). Some PSGs are however expressed in these cells, such as *CDC25C*, *FRMD4B*, *NHSL1, NUSAP1*, and *PLEKHA5*.

In single-cell transcriptomic studies of the mouse cerebellum (Carter et al. 2018), we found that cells expressing cilium marker genes, such as the dynein light chain roadblock-type 2 (*DYNLRB2*) and the meiosis/spermiogenesis associated 1 (*MEIG1*), were the principal cells with higher levels of PSG expression (Fig. 4C; Supplemental Table S5). Those “ciliated cells” were not anatomically identified in the cerebellum (Carter et al. 2018), but their associated cilium markers were found to be expressed at the site of the cerebellar granule cells (Lein et al. 2007). These cells may, therefore, be a subtype of granule neurons involved in cerebellar function. The PSGs expressed in these ciliated cells code for the tubulin tyrosine ligase-like family, member 6 (*TTLL6*), *TOP3A*, the dynein cytoplasmic 2 light intermediate chain 1 (*DYNC2LI1*), and LCA5, coding for a component of the axoneme of ciliated cells. Some of these PSGs are also involved in human brain diseases such as microcephaly, macrocephaly, and Joubert syndrome (Fig. 4D, and below).

Finally, we assessed the potential association with brain functions, by extracting 19,244 brain imaging results from 315 fMRI-BOLD studies (*T* and *Z*-score maps) (for the complete list, see Supplemental Table S6) from NeuroVault (Gorgolewski et al. 2015) and comparing the spatial patterns observed with the patterns of gene expression in the Allen Brain Atlas (Hawrylycz et al. 2012; Gorgolewski et al. 2014). The correlation between brain activity and PSG expression was stronger in subcortical structures than in the cortex (Wilcoxon rc = 0.14, *P* = 2.5 × 10^−248^). The brain activity maps that correlate with the expression pattern of the PSGs (Supplemental Table S7) were enriched in social tasks (empathy, emotion recognition, theory of mind, language; Fisher's exact test *P* = 2.9 × 10^−20^, OR = 1.72, CI_95%_ = [1.53, 1.93]). We also observed this enrichment for expression pattern of the SCGs (Fisher's exact test *P* = 1.2 × 10^−12^, OR = 1.16, CI_95%_ = [1.11, 1.22]); however, it was significantly less enriched than for the expression pattern of PSGs (Fisher's exact test *P* = 0.0004, OR = 0.83, CI_95%_ = [0.75, 0.92]).

### Positively selected genes and their relationship to brain disorders

Our systematic analysis revealed that SCGs were more associated with brain diseases or traits than PSGs (Fig. 3B), particularly for intellectual disability (*P* = 8.13 × 10^−6^, OR = 0.34 CI_95%_ = [0.21, 0.56], Bonferroni-corrected) and autism (*P* = 0.0005, OR = 0.26, CI_95%_ = [0.11, 0.59], Bonferroni-corrected). We also identified 42 high PSGs associated (based on OMIM and HPO data) with several human diseases or conditions, such as micro/macrocephaly, autism, or dyslexia (Supplemental Table S4).

**Figure 5.**
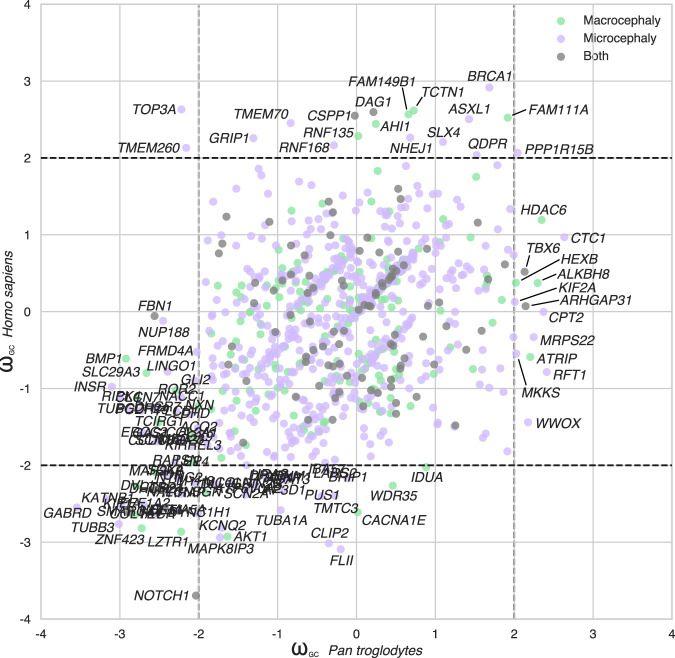
Evolution of the protein-coding genes associated with microcephaly or macrocephaly in humans. Scatter plots comparing ω_GC12_ between *Homo sapiens* and *Pan troglodytes* for the microcephaly- and macrocephaly-associated genes.

A comparison of humans and chimpanzees with our common primate ancestor revealed several protein-coding genes associated with micro/macrocephaly with different patterns of evolution in humans and chimpanzees (Fig. 5). Some genes displayed a divergence specifically in the hominin lineage (*AHI1*, *ASXL1*, *BRCA1*, *CSPP1*, *DAG1*, *FAM111A*, *FAM149B1*, *GRIP1*, *NHEJ1*, *QDPR*, *RNF135*, *RNF168*, *SLX4*, *TCTN1*, *TMEM70*, *TMEM260*, and *TOP3A*) or in the chimpanzee (*ALKBH8*, *ARHGAP31*, *ATRIP*, *CPT2*, *CTC1*, *HDAC6*, *HEXB*, *KIF2A*, *MKKS*, *MRPS22*, *RFT1*, *TBX6*, and *WWOX*).

**Figure 6.**
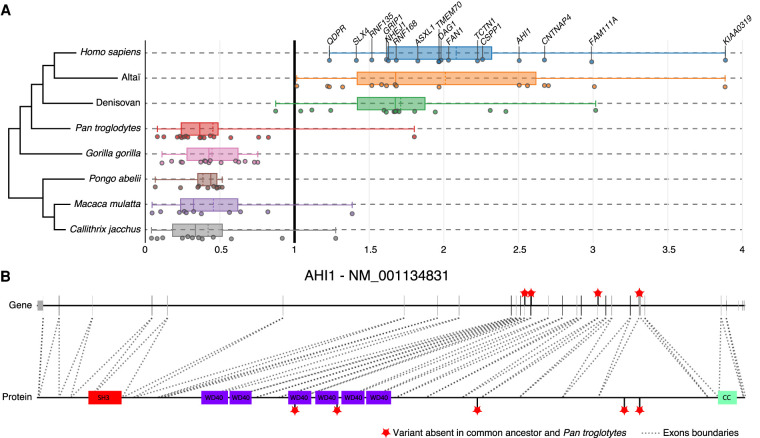
Examples of brain disorder–associated protein-coding genes displaying specific divergence in hominins during primate evolution. (*A*) Representation of 16 genes with *d*_N_/*d*_S_ > 1 in *Homo sapiens* and archaic hominins but *d*_N_/*d*_S_ < 1 for other primates. (*B*) Representation of hominin-specific nonsynonymous variants of the *AHI1* gene, showing the correspondence with the protein (dotted lines indicate exons); note how two variants lie within the WD40 functional domains. Red stars indicate variants (CADD > 5) relative to the ancestor present in *Homo sapiens*, Neanderthals, and Denisovans, but not in *Pan troglodytes*. (WD40) WD40 repeat; (SH3) SRC homology 3; (CC) coiled coils.

We also identified PSGs associated with communication disorders, such as autism (*CNTNAP4, AHI1, FAN1*, *SNTG2*, and *GRIP1*) and dyslexia (*KIAA0319*). These genes diverged from the common primate ancestor only in the hominin lineage and were under strong selective constraint in all other taxa (Fig. 6A,B). They all have roles relating to neuronal connectivity (neuronal migration and synaptogenesis) and, within the human brain, were more specifically expressed in the cerebellum, except for *GRIP1*, which was expressed almost exclusively in the cortex.

The dyslexia susceptibility gene *KIAA0319*, encoding a protein involved in axon growth inhibition (Paracchini et al. 2006; Franquinho et al. 2017), is one of the PSGs under the strongest positive selection in humans relative to the common primate ancestor (raw *d*_N_/*d*_S_ = 3.9; nine nonsynonymous vs. one synonymous mutations in *Homo sapiens* compared to the common primate ancestor). The role of *KIAA0319* in dyslexia remains a matter of debate, but its rapid evolution in the hominoid lineage warrants further genetic and functional studies.

Finally, several PSGs display very high levels of positive selection in *Homo sapiens*, but their functions or association with disease remain unknown. For example, the zinc finger protein ZNF491 (raw *d*_N_/*d*_S_ = 4.7; 14 nonsynonymous vs. one synonymous mutation in *Homo sapiens* compared to the common primate ancestor) is specifically expressed in the cerebellum and is structurally similar to a chromatin remodeling factor, but its biological role remains to be determined. Another example is the *CCP110* gene, encoding a centrosomal protein resembling ASPM, but not associated with a disease. Its function suggests that this PSG would be a compelling candidate for involvement in microcephaly in humans. A complete list of the brain SCGs and PSGs is available in Supplemental Table S4 and on the companion website.

## Discussion

### Positively selected genes and brain size in primates

Several protein-coding genes are thought to have played a significant role in the increase in brain size in humans. Some of these genes, such as *ARHGAP11B, SRGAP2C,* and *NOTCH2NLA* (Suzuki et al. 2018), are specific to humans, having recently been duplicated (Dennis and Eichler 2016). Other studies have suggested that a high degree of positive selection in genes involved in micro/macrocephaly may have contributed to the substantial change in brain size during primate evolution (Dorus et al. 2004; Hayward 2004). Several of these genes, such as *ASPM* (Mekel-Bobrov et al. 2005) and *MCPH1* (Evans et al. 2005), seem to have evolved more rapidly in humans. However, the adaptive nature of the evolution of these genes has been called into question (Yu et al. 2007), and neither of these two genes were on the PSG list in our analysis (their raw *d*_N_/*d*_S_ value are below 0.8).

Conversely, our systematic detection approach identified the genes under the strongest positive selection in humans for micro/macrocephaly, the top 10 such genes being *AHSG*, *ASXL1, BRCA1*, *CSPP1, DAG1, FAM111A, FAM149B1, RNF168, TMEM70,* and *TOP3A*. This list of PSGs associated with micro/macrocephaly in humans can be used to select the best candidate human-specific gene/variants for further genetic and functional analyses, to improve estimates of their contribution to the emergence of anatomic difference between humans and other primates.

As previously shown, our systematic analysis confirms that the major susceptibility gene for breast cancer *BRCA1* is under strong positive selection (Lou et al. 2014). BRCA1 is a DNA damage response protein that repairs double-strand breaks in DNA. Heterozygous *BRCA1* mutations increase the risk of breast cancer but can also cause neuronal migration defects (Eccles et al. 2005). In sporadic cases, homozygous *BRCA1* mutations lead to Fanconi anemia with microcephaly (Mehmet et al. 2016). Several other DNA damage response proteins (Arcas et al. 2014), which are binding partners of BRCA1 (such as SLX4, TOP3A, RNF168, and MCPH1) are also associated with microcephaly. How *BRCA1* mutations cause microcephaly in humans remains largely unknown. However, in the mouse, *Brca1* mutations strongly reduce the size of the cerebral cortex by affecting the cellular polarity of neural progenitors and preventing the apoptosis of early cortical neuron progenitors (Pulvers and Huttner 2009; Pao et al. 2014). Upper-most cortical layers are not reduced upon *Brca1* ablation in mice, and this is consistent with the low levels of apoptosis found in late progenitors and the neurons derived from there. Our analysis of the single-cell RNA-seq data from the human cortex indicates that excitatory neurons from layers 3 and 5 express PSG more than expected, including *BRCA1* and several of its binding partners associated with DNA damage response and microcephaly, such as *TOP3A*, *RNF168,* and *NHEJ1*. Further analyses on the role of these genes, which are currently known for their DNA damage response, might shed some light on primate brain evolution.

In addition to brain size, some of the micro/macrocephaly PSGs may have contributed to differences in other morphological features, such as skeleton development. For example, the PSG *FAM111A* (raw *d*_N_/*d*_S_ = 2.99; seven nonsynonymous vs. one synonymous mutation in *Homo sapiens* compared to the common primate ancestor) and *ASXL1* (raw *d*_N_/*d*_S_ = 1.83; 12 nonsynonymous vs. three synonymous mutations in *Homo sapiens* compared to the common primate ancestor) are associated with macrocephaly and microcephaly, respectively. Patients with dominant mutations of *FAM111A* are diagnosed with Kenny-Caffey syndrome (KCS). They display impaired skeletal development, with small dense bones, short stature, primary hypoparathyroidism with hypocalcemia, and a prominent forehead (Unger et al. 2013). FAM111A is a binding partner of BRCA1 and plays a role in DNA damage response, but this protein seems to be also crucial to a pathway governing parathyroid hormone production, calcium homeostasis, and skeletal development and growth. In contrast, patients with dominant mutations of *ASXL1* are diagnosed with Bohring-Opitz syndrome, a malformation syndrome characterized by severe intrauterine growth retardation, intellectual disability, trigonocephaly, hirsutism, and flexion of the elbows and wrists with a deviation of the wrists and metacarpophalangeal joints (Hoischen et al. 2011). *ASXL1* encodes a chromatin protein required to maintain both the activation and silencing of homeotic genes.

Three genes (*AHI1, CSPP1,* and *TCTN1*) in the top 10 of the PSGs associated with human brain diseases, with raw *d*_N_/*d*_S_ > 2, are required for both cortical and cerebellar development in humans. They are also associated with Joubert syndrome, a recessive disease characterized by agenesis of the cerebellar vermis and difficulties coordinating movements. *AHI1* is a positive modulator of classical WNT/ciliary signaling. *CSPP1* is involved in cell cycle–dependent microtubule organization, and *TCTN1* is a regulator of Hedgehog during development.

*AHI1* was previously identified as a gene subject to positive selection during the evolution of the human lineage (Ferland et al. 2004; Gould and Walter 2004), but to our knowledge, neither *CSPP1* nor *TCTN1* has previously been described as diverging during primate evolution. It has been suggested that the accelerated evolution of *AHI1* required for ciliogenesis and axonal growth may have played a role in the development of unique motor capabilities, such as bipedalism, in humans (Hayward 2004). Our findings provide further support for the accelerated evolution of a set of genes associated with ciliogenesis.

### The possible link between a change in the genetic makeup of the cerebellum and the evolution of human cognition

The emergence of a large cortex was undoubtedly an essential step for human cognition, but other parts of the brain, such as the cerebellum, may also have made significant contributions to both motricity and cognition. In this study, we showed that the protein-coding genes expressed in the cerebellum were among the most conserved in humans. However, we also identified a set of PSGs with relatively strong expression in the cerebellum or for which mutations affected the cerebellar function. As discussed above, several PSGs are associated with Joubert syndrome, including *AHI1, CSPP1,* and *TCTN1,* and are essential for cerebellar development. Furthermore, the PSGs expressed in the brain and under the highest positive selection include *CNTNAP4, FAN1, SNTG2*, and *KIAA0319*, which also display high levels of expression in the cerebellum and have been associated with communication disorders, such as autism and dyslexia. Finally, the choroid plexus expressed more PSGs than expected and is known to play the role of a paracrine gland to produce the retinoic acid necessary for cerebellum development (Yamamoto et al. 1996).

In humans, the cerebellum is associated with higher cognitive functions, such as visuospatial skills, the planning of complex movements, procedural learning, attention switching, and sensory discrimination (Koziol et al. 2012). It plays a crucial role in temporal processing (Rao et al. 2001) and the anticipation and control of behavior through both implicit and explicit mechanisms (Koziol et al. 2012). A change in the genetic makeup of the cerebellum would, therefore, be expected to have been of great advantage for the emergence of the specific features of human cognition.

Despite this possible link between the cerebellum and the emergence of human cognition, much less attention has been paid to this part of the brain than to the cortex, on which most of the functional studies investigating the role of human-specific genes/variants have focused. For example, *SRGAP2C* expression is considerably high in the human cerebellum, but the ectopic expression of this gene has been studied in mouse cortex (Charrier et al. 2012; Dennis et al. 2012), in which it triggers human-like neuronal characteristics, such as an increase in dendritic spine length and density. We thus suggest that an exploration of human genes/variants specifically associated with the development and functioning of the cerebellum might shed new light on the evolution of human cognition.

### Limitations

The present results have potential limits in their interpretations. Sources of error in the alignments (e.g., false orthologous, segmental duplications, errors in ancestral sequence reconstruction) are still possible and can result in inflated *d*_N_/*d*_S_. The *d*_N_/*d*_S_ method is not suited for comparing very closely related species and therefore, differences between *Neanderthal*, *Denisovan*, and *Homo sapiens* must be taken with care. Moreover, methods to estimate the evolution of proteins are expected to give downwardly biased estimates (Eyre-Walker and Keightley 2009). However, our GC12 normalization has already proved to correct for most of those biases in systematic analyses (Kapheim et al. 2015), and our raw *d*_N_/*d*_S_ values highly correlate with other independent studies on primates (Nielsen et al. 2005; Biswas et al. 2016). Moreover, for the enrichment analyses, we used bootstrapping techniques to better control for potential biases induced by differences in GC content and gene length, especially for genes implicated in brain disorders (Zylka et al. 2015). Finally, our data are openly available on the companion website and allow checking at the variant level which amino acids changed.

### Perspectives

Our systematic analysis of protein sequence diversity confirmed that protein-coding genes relating to brain function are among the most highly conserved in the human genome. The set of PSGs identified here may have played specific roles in the evolution of human cognition, by modulating brain size, neuronal migration, and synaptic physiology, but further genetic—including detailed analyses of all species branches—and functional studies would shed new light on the role of these genes. Beyond the brain, this resource will also be useful for estimating the evolutionary pressure acting on genes related to other biological pathways, particularly those displaying signs of positive selection during primate evolution, such as the reproductive and immune systems.

## Methods

### Genetic sequences

#### Alignments with the reference genome

We collected sequences and reconstructed sequence alignments with the reference human genome version hg19 (release 19, GRCh37.p13). For the primate common ancestor sequence, we used the Ensembl 6-way Enredo-Pecan-Ortheus (EPO) (Paten et al. 2008) multiple alignments v71, related to *Homo sapiens* (hg19), chimpanzee (panTro4), gorilla (gorGor3), orangutan (ponAbe2), rhesus macaque (rheMac3), and marmoset (calJac3). For the two ancestral hominins, Altai, and Denisovan, we integrated variants detected by Castellano et al. (2014) into the standard hg19 sequence (http://cdna.eva.mpg.de/neandertal/; date of access July 3, 2014). Finally, we used the whole-genome alignment of all the primates used in the 6-EPO from the UCSC website (http://hgdownload.soe.ucsc.edu/downloads.html; date of access online August 13, 2015). All the PSGs had their protein sequence deduced from our analysis compared manually to the one in the protein database. All variants matched, and we did not find any alignment artifact. The core annotations used for our study were not available for the GRCh38 version of the human genome when we started this project. Because one of the biggest improvements in GRCh38 is the annotation of the centromere regions (Guo et al. 2017), a switch from GRCh37 to GRCh38 would not affect our conclusions. Moreover, regarding the coding regions of the human genome, the number of nonsynonymous detected by GRCh38 (*N* = 22,796 SNVs) is very similar to GRCh37's (*N* = 22,622 SNVs) (Guo et al. 2017, Table 3).

#### VCF annotation

We combined the VCF file from Castellano et al. (2014) with the VCF files generated from the ancestor and primate sequence alignments. The global VCF was annotated with ANNOVAR (version of June 2015) (Wang et al. 2010), using the following databases: refGene, cytoBand, genomicSuperDups, esp6500siv2_all, 1000g2014oct_all, 1000g2014oct_afr, 1000g2014oct_eas, 1000g2014oct_eur, avsnp142, ljb26_all, gerp++elem, popfreq_max, exac03_all, exac03_afr, exac03_amr, exac03_eas, exac03_fin, exac03_nfe, exac03_oth, exac03_sas. We also used the ClinVar database (https://ncbi.nlm.nih.gov/clinvar/; date of access February 3, 2016).

### ω_GC12_ calculation

Once all the alignments had been collected, we extracted the consensus coding sequences (CCDS) of all protein-coding genes referenced in Ensembl BioMart GRCh37, according to the HGNC (date of access May 5, 2015) and NCBI Consensus CDS protein set (date of access August 10, 2015). We calculated the number of nonsynonymous mutations *N*, the number of synonymous mutations *S*, the ratio of the number of nonsynonymous mutations per nonsynonymous site *d*_N_, the number of synonymous mutations per synonymous site *d*_S_, and their ratio *d*_N_/*d*_S_ (also called ω) between all taxa and the ancestor, using the yn00 algorithm implemented in PamL software (Yang 2007). We avoided infinite and null results by calculating a corrected version of *d*_N_/*d*_S_. If S was null, we set its value to 1 to avoid having zero as the numerator. The obtained values were validated through the replication of a recent systematic estimation of *d*_N_/*d*_S_ between *Homo sapiens* and two great apes (*Pan troglodytes* and *Pongo abelii*; Pearson's *r* > 0.8, *P* < 0.0001) (Supplemental Fig. S2; Biswas et al. 2016). Finally, we obtained our ω_GC12_ value by correcting for the GC12 content of the genes with a generalized linear model and by calculating a *Z*-score for each taxon (Kapheim et al. 2015). GC content has been associated with biases in mutation rates, particularly in primates (Galtier et al. 2009) and humans (Kostka et al. 2012). We retained only the 11,667 genes with 1:1 orthologs in primates (extracted for GRCh37.p13 with Ensembl BioMart; accessed online February 27, 2017).

### Gene sets

We used different gene sets, starting at the tissue level and then focusing on the brain and key pathways. For body tissues, we used Illumina Body Map 2.0 RNA-seq data, corresponding to 16 human tissue types: adrenal, adipose, brain, breast, colon, heart, kidney, liver, lung, lymph, ovary, prostate, skeletal muscle, testes, thyroid, and white blood cells (for more information: https://www.ebi.ac.uk/gxa/experiments/E-MTAB-513; data preprocessed with Cufflinks, accessed May 5, 2015, at http://cureffi.org). We also used the microarray data set of Su et al. (2004) (Human U133A/GNF1H Gene Atlas, accessed May 4, 2015, at http://biogps.org). Finally, we also replicated our results with recent RNA-seq data from The GTEx Consortium (2015) (https://www.gtexportal.org/home/).

For the brain, we used the data set of Su et al. (2004) and the Human Protein Atlas data (accessed November 7, 2017, at https://www.proteinatlas.org). For analysis of the biological pathways associated with the brain, we used KEGG (accessed February 25, 2015, at https://www.genome.jp/kegg/), synaptic genes curated by the group of Danielle Posthuma at Vrije Universiteit (accessed September 1, 2014, at https://ctg.cncr.nl/software/genesets) (Ruano et al. 2010; Lips et al. 2012), and mass spectrometry data from Loh (2016). Finally, for the diseases associated with the brain, we combined gene sets generated from Human Phenotype Ontology (accessed August 14, 2020, at https://hpo.jax.org) including OMIM annotation (https://omim.org) and curated lists: the 65 risk genes proposed by Sanders et al. (2015) (TADA), the candidate genes for autism spectrum disorders from SFARI (accessed July 17, 2015, at https://gene.sfari.org), the Developmental Brain Disorder (DBD) (accessed July 12, 2016, at https://geisingeradmi.org/care-innovation/studies/dbd-genes/), and Cancer Census (accessed November 24, 2016, at https://cancer.sanger.ac.uk/census) data. Note that the combination of HPO and OMIM is the most exhaustive, making it possible to avoid missing potential candidate genes, but this combination does not identify specific associations.

SynGO was generously provided by Matthijs Verhage (accessed on January 11, 2019). This ontology is a consistent, evidence-based annotation of synaptic gene products developed by the SynGO consortium (2015–2017) in collaboration with the GO consortium. It extends the existing Gene Ontology (GO) of the synapse and follows the same dichotomy between biological processes (BP) and cellular components (CC).

For single-cell transcriptomics data sets, we identified the genes specifically highly expressed in each cell type, following the same strategy as used for the other RNA-seq data sets. The single-cell data for the developing human cortex were kindly provided by Maximilian Haeussler (available at https://cells.ucsc.edu; accessed on October 30, 2018). The single-cell transcriptional atlas data for the developing murine cerebellum (Carter et al. 2018) were kindly provided by Robert A. Carter (accessed on January 29, 2019). For each cell type, we combined expression values cross all available replicates, to guarantee a high signal-to-noise ratio. We then calculated the values for the associated genes in *Homo sapiens* according to the paralogous correspondence between humans and mice (Ensembl BioMart accessed on February 23, 2019).

### Gene nomenclature

We extracted all the Entrez ID of the protein-coding genes for GRCh37 from Ensembl BioMart. We used the HGNC database to recover their symbols. For the 46 unmapped genes, we searched the NCBI database manually for the official symbol.

### McDonald–Kreitman test, neutrality index, and Direction of Selection

We assessed the possible fixation of variants in the *Homo sapiens* population by first calculating the relative ratio of nonsynonymous to synonymous polymorphism (*p*_N_/*p*_S_) from the 1000 Genomes VCF for all SNPs, for SNPs with a minor allele frequency (MAF) <1% and <5%. SNPs were annotated with ANNOVAR across 1000 Genomes Project (ALL+5 ethnicity groups), ESP6500 (ALL+2 ethnicity groups), ExAC (ALL+7 ethnicity groups), and CG46 (for more details, see http://annovar.openbioinformatics.org/en/latest/user-guide/filter/#popfreqmax-and-popfreqall-annotations). The polymorphism ratio (*p*_N_/*p*_S_) allowed us to take into account the constraint on nonsynonymous sites and thus increase the power of detecting positive selection (Salvador-Martínez et al. 2018). We indeed normalized the divergence ratio (*d*_N_/*d*_S_) using the McDonald–Kreitman (MK) test, that is, calculating the neutrality index (NI) as the ratio of raw *p*_N_/*p*_S_ and *d*_N_/*d*_S_ values (McDonald and Kreitman 1991). We considered the PSG to be fixed in the population when NI < 1. We also confirmed with a new statistic for evolutionary measure: the Direction of Selection (DoS) = *D*_n_/(*D*_n_ + *D*_s_) − *P*_n_/(*P*_n_ + *P*_s_) (Stoletzki and Eyre-Walker 2011) that all divergent genes with NI < 0 had a DoS < 0 (Supplemental Fig. S8).

### NeuroVault analyses

We used the NeuroVault website (Gorgolewski et al. 2015) to collect 19,244 brain imaging results from fMRI-BOLD studies (*T* and *Z*-score maps) and their correlation with the gene expression data (Gorgolewski et al. 2014) of the Allen Brain Atlas (Hawrylycz et al. 2012). The gene expression data of the Allen Brain Atlas were normalized and projected into the MNI152 stereotactic space used by NeuroVault, using the spatial coordinates provided by the Allen Brain Institute. An inverse relationship between cortical and subcortical expression dominated the pattern of expression for many genes. We thus calculated the correlations for the cortex and subcortical structures separately.

### Allen brain data

We downloaded the Allen Brain Atlas microarray-based gene data and multiple cortical areas: Smart-seq from the Allen Brain website (accessed on July, 2020, at http://www.brain-map.org). Microarray data were available for six adult brains; the right hemisphere was missing for three donors, so we considered only the left hemisphere for our analyses. For each donor, we averaged probes targeting the same gene and falling in the same brain area. We then subjected the data to log normalization and calculated *Z*-scores: across the 20,787 genes for each brain region to obtain expression levels; and across the 212 brain areas for each gene to obtain expression specificity. For genes with more than one probe, we averaged the normalized values over all probes available. The Smart-seq data set followed a similar preprocessing and led to expression level and specificity of 32,165 genes across 363 cell types.

As a complementary data set, we also used a mapping of the Allen Brain Atlas onto the 68 brain regions of the FreeSurfer atlas (French and Paus 2015) (accessed on April 4, 2017, at https://figshare.com/articles/A_FreeSurfer_view_of_the_cortical_transcriptome_generated_from_the_Allen_Human_Brain_Atlas/1439749). The expression and specificity measure were used for the 3D visualization in the companion website.

### Statistics

#### Enrichment analyses

We first calculated a two-way hierarchical clustering on the normalized *d*_N_/*d*_S_ values (**ω**_GC_) across the whole genome (Fig. 1B). Of note, 11,667 genes were included in the analysis to ensure medium-quality coverage for *Homo sapiens*, Neanderthals, Denisovans, and *Pan troglodytes* (Supplemental Fig. S1). According to 30 clustering indices (Charrad et al. 2014), the best partitioning in terms of evolutionary pressure was into two clusters of genes: SCGs (*N* = 4825; in HS, mean = −0.88 median = −0.80 SD = 0.69) and PSGs (*N* = 6842; in HS, mean = 0.60 median = 0.48 SD = 0.63). For each cluster, we calculated the enrichment in biological functions in Cytoscape (Shannon et al. 2003) with the BINGO plugin (Maere et al. 2005). We used all 11,667 genes as the background. We eliminated redundancy by first filtering out all the statistically significant Gene Ontology (GO) terms associated with fewer than 10 or more than 1000 genes, and then combining the remaining genes with the EnrichmentMap plugin (Merico et al. 2010). We used a *P*-value cutoff of 0.005, an FDR *Q*-value cutoff of 0.05, and a Jaccard coefficient of 0.5.

For the cell type–specific expression analysis (CSEA) (Xu et al. 2014), we used the CSEA method with the online tool http://genetics.wustl.edu/jdlab/csea-tool-2/. This method associates gene lists with brain expression profiles across cell types, regions, and time periods.

#### Wilcoxon and rank-biserial correlation

We investigated the extent to which each gene set was significantly more under positive or constraint selection than expected by chance by performing Wilcoxon tests on the normalized *d*_N_/*d*_S_ values (**ω**_GC_) for the genes in the set against zero (the mean value for the genome). We quantified effect size by matched pairs rank-biserial correlation, as described by Kerby (2014). Following nonparametric Wilcoxon signed-rank tests, the rank-biserial correlation was evaluated as the difference between the proportions of negative and positive ranks over the total sum of ranks as follows:

$$rc=\frac{\sum r_{+}-\sum r_{-}}{\sum r_{+}+\sum r_{-}}=f-u.$$

It corresponds to the difference between the proportion of observations consistent with the hypothesis (*f*) minus the proportion of observations contradicting the hypothesis (*u*), thus representing an effect size. Like other correlational measures, its value ranges from −1 to +1, with a value of zero indicating no relationship. In our case, a negative rank-biserial correlation corresponds to a gene set in which more genes have negative *ω*_*GC*_ values than positive values, revealing a degree of conservation greater than the mean for all genes (i.e., *ω*_*GC*_ = 0). Conversely, a positive rank-biserial correlation corresponds to a gene set that is more under positive selection than expected by chance (i.e., taking randomly the same number of genes across the whole genome; correction for the potential biases for GC content and CDS length are done at the bootstrap level). All statistics relating to Figures 1D and 2, A and B, are summarized in Supplemental Table S3. All those relating to Figure 4 are summarized in Supplemental Table S5.

#### Validation by resampling

We also used bootstrapping to correct for potential bias in the length of the coding sequence or the global specificity of gene expression (Tau, see the methods in Kryuchkova-Mostacci and Robinson-Rechavi 2016). For each of the 10,000 permutations, we randomly selected the same number of genes as for the sample of genes from the complete set of genes for which *d*_N_/*d*_S_ was not missing. We corrected for CCDS length and GC content by bootstrap resampling. We estimated significance, to determine whether the null hypothesis could be rejected, by calculating the number of bootstrap draws (*B*_*i*_) falling below and above the observed measurement (*m*). The corresponding empirical *P*-value was calculated as follows:

$$p=2\times \min\left(\frac{1+\sum_{i}B_{i}\geq m}{N+1},\frac{1+\sum_{i}B_{i}\leq m}{N+1}\right).$$

## Data access

All the data and code supporting the findings of this study are available from our resource website (https://genevo.pasteur.fr) and as Supplemental Material.

### Competing interest statement

The authors declare no competing interests.

## Supplementary Material

Supplemental Material

## References

[GR262113DUMC1] Arcas A, Fernández-Capetillo O, Cases I, Rojas AM. 2014. Emergence and evolutionary analysis of the human DDR network: implications in comparative genomics and downstream analyses. Mol Biol Evol 31: 940–961. 10.1093/molbev/msu046 24441036 PMC3969565

[GR262113DUMC2] Atkinson EG, Audesse AJ, Palacios JA, Bobo DM, Webb AE, Ramachandran S, Henn BM. 2018. No evidence for recent selection at *FOXP2* among diverse human populations. Cell 174: 1424–1435.e15. 10.1016/j.cell.2018.06.048 30078708 PMC6128738

[GR262113DUMC3] Balsters JH, Cussans E, Diedrichsen J, Phillips KA, Preuss TM, Rilling JK, Ramnani N. 2010. Evolution of the cerebellar cortex: the selective expansion of prefrontal-projecting cerebellar lobules. Neuroimage 49: 2045–2052. 10.1016/j.neuroimage.2009.10.045 19857577 PMC6436533

[GR262113DUMC4] Biswas K, Chakraborty S, Podder S, Ghosh TC. 2016. Insights into the dN/dS ratio heterogeneity between brain specific genes and widely expressed genes in species of different complexity. Genomics 108: 11–17. 10.1016/j.ygeno.2016.04.004 27126306

[GR262113DUMC5] Brazel CY, Romanko MJ, Rothstein RP, Levison SW. 2003. Roles of the mammalian subventricular zone in brain development. Prog Neurobiol 69: 49–69. 10.1016/S0301-0082(03)00002-9 12637172

[GR262113DUMC6] Calarco JA, Xing Y, Cáceres M, Calarco JP, Xiao X, Pan Q, Lee C, Preuss TM, Blencowe BJ. 2007. Global analysis of alternative splicing differences between humans and chimpanzees. Genes Dev 21: 2963–2975. 10.1101/gad.1606907 17978102 PMC2049197

[GR262113DUMC7] Camp JG, Badsha F, Florio M, Kanton S, Gerber T, Wilsch-Bräuninger M, Lewitus E, Sykes A, Hevers W, Lancaster M, 2015. Human cerebral organoids recapitulate gene expression programs of fetal neocortex development. Proc Natl Acad Sci 112: 15672–15677. 10.1073/pnas.1520760112 26644564 PMC4697386

[GR262113DUMC8] Carter RA, Bihannic L, Rosencrance C, Hadley JL, Tong Y, Phoenix TN, Natarajan S, Easton J, Northcott PA, Gawad C. 2018. A single-cell transcriptional atlas of the developing murine cerebellum. Curr Biol 28: 2910–2920.e2. 10.1016/j.cub.2018.07.062 30220501

[GR262113DUMC9] Castellano S, Parra G, Sanchez-Quinto FA, Racimo F, Kuhlwilm M, Kircher M, Sawyer S, Fu Q, Heinze A, Nickel B, 2014. Patterns of coding variation in the complete exomes of three Neandertals. Proc Natl Acad Sci 111: 6666–6671. 10.1073/pnas.1405138111 24753607 PMC4020111

[GR262113DUMC10] Changeux JP. 2017. Climbing brain levels of organisation from genes to consciousness. Trends Cogn Sci 21: 168–181. 10.1016/j.tics.2017.01.004 28161289

[GR262113DUMC11] Charrad M, Ghazzali N, Boiteau V, Niknafs A. 2014. NbClust: an R package for determining the relevant number of clusters in a data set. J Stat Softw 61: 1–36. 10.18637/jss.v061.i06

[GR262113DUMC12] Charrier C, Joshi K, Coutinho-Budd J, Kim J-E, Lambert N, de Marchena J, Jin W-L, Vanderhaeghen P, Ghosh A, Sassa T, 2012. Inhibition of SRGAP2 function by its human-specific paralogs induces neoteny during spine maturation. Cell 149: 923–935. 10.1016/j.cell.2012.03.034 22559944 PMC3357949

[GR262113DUMC13] Dennis MY, Eichler EE. 2016. Human adaptation and evolution by segmental duplication. Curr Opin Genet Dev 41: 44–52. 10.1016/j.gde.2016.08.001 27584858 PMC5161654

[GR262113DUMC14] Dennis MY, Nuttle X, Sudmant PH, Antonacci F, Graves TA, Nefedov M, Rosenfeld JA, Sajjadian S, Malig M, Kotkiewicz H, 2012. Evolution of human-specific neural *SRGAP2* genes by incomplete segmental duplication. Cell 149: 912–922. 10.1016/j.cell.2012.03.033 22559943 PMC3365555

[GR262113DUMC15] Dennis MY, Harshman L, Nelson BJ, Penn O, Cantsilieris S, Huddleston J, Antonacci F, Penewit K, Denman L, Raja A, 2017. The evolution and population diversity of human-specific segmental duplications. Nat Ecol Evol 1: 69. 10.1038/s41559-016-0069 28580430 PMC5450946

[GR262113DUMC16] Dorus S, Vallender EJ, Evans PD, Anderson JR, Gilbert SL, Mahowald M, Wyckoff GJ, Malcom CM, Lahn BT. 2004. Accelerated evolution of nervous system genes in the origin of homo sapiens. Cell 119: 1027–1040. 10.1016/j.cell.2004.11.040 15620360

[GR262113DUMC17] Dunbar RIM, Shultz S. 2017. Why are there so many explanations for primate brain evolution? Phil Trans R Soc B 372: 20160244. 10.1098/rstb.2016.0244 28673920 PMC5498304

[GR262113DUMC18] Eccles D, Bunyan D, Barker S, Castle B. 2005. BRCA1 mutation and neuronal migration defect: implications for chemoprevention. J Med Genet 42: e42. 10.1136/jmg.2004.028084 15863656 PMC1736045

[GR262113DUMC19] Enard W, Przeworski M, Fisher SE, Lai CSL, Wiebe V, Kitano T, Monaco AP, Pääbo S. 2002. Molecular evolution of *FOXP2*, a gene involved in speech and language. Nature 418: 869–872. 10.1038/nature01025 12192408

[GR262113DUMC20] Enard W, Gehre S, Hammerschmidt K, Hölter SM, Blass T, Somel M, Brückner MK, Schreiweis C, Winter C, Sohr R, 2009. A humanized version of Foxp2 affects cortico-basal ganglia circuits in mice. Cell 137: 961–971. 10.1016/j.cell.2009.03.041 19490899

[GR262113DUMC21] Evans PD, Gilbert SL, Mekel-Bobrov N, Vallender EJ, Anderson JR, Vaez-Azizi LM, Tishkoff SA, Hudson RR, Lahn BT. 2005. Microcephalin, a gene regulating brain size, continues to evolve adaptively in humans. Science 309: 1717–1720. 10.1126/science.1113722 16151009

[GR262113DUMC22] Eyre-Walker A, Keightley PD. 2009. Estimating the rate of adaptive molecular evolution in the presence of slightly deleterious mutations and population size change. Mol Biol Evol 26: 2097–2108. 10.1093/molbev/msp119 19535738

[GR262113DUMC23] Ferland RJ, Eyaid W, Collura RV, Tully LD, Hill RS, Al-Nouri D, Al-Rumayyan A, Topcu M, Gascon G, Bodell A, 2004. Abnormal cerebellar development and axonal decussation due to mutations in *AHI1* in Joubert syndrome. Nat Genet 36: 1008–1013. 10.1038/ng1419 15322546

[GR262113DUMC24] Florio M, Albert M, Taverna E, Namba T, Brandl H, Lewitus E, Haffner C, Sykes A, Wong FK, Peters J, 2015. Human-specific gene *ARHGAP11B* promotes basal progenitor amplification and neocortex expansion. Science 347: 1465–1470. 10.1126/science.aaa1975 25721503

[GR262113DUMC25] Franquinho F, Nogueira-Rodrigues J, Duarte JM, Esteves SS, Carter-Su C, Monaco AP, Molnár Z, Velayos-Baeza A, Brites P, Sousa MM. 2017. The dyslexia-susceptibility protein KIAA0319 inhibits axon growth through Smad2 signaling. Cereb Cortex 27: 1732–1747. 10.1093/cercor/bhx023 28334068 PMC5905272

[GR262113DUMC26] French L, Paus T. 2015. A FreeSurfer view of the cortical transcriptome generated from the Allen Human Brain Atlas. Front Neurosci 9: 323. 10.3389/fnins.2015.00323 26441498 PMC4584957

[GR262113DUMC27] Frith C, Dolan R. 1996. The role of the prefrontal cortex in higher cognitive functions. Cogn Brain Res 5: 175–181. 10.1016/S0926-6410(96)00054-7 9049084

[GR262113DUMC28] Galtier N, Duret L, Glémin S, Ranwez V. 2009. GC-biased gene conversion promotes the fixation of deleterious amino acid changes in primates. Trends Genet 25: 1–5. 10.1016/j.tig.2008.10.011 19027980

[GR262113DUMC29] Gorgolewski KJ, Fox AS, Chang L, Schäfer A, Arélin K, Burmann I, Sacher J, Margulies DS, Gorgolewski KJ, Fox AS, 2014. Tight fitting genes: finding relations between statistical maps and gene expression patterns. F1000Res 5: 1607. 10.7490/f1000research.1097120.1

[GR262113DUMC30] Gorgolewski KJ, Varoquaux G, Rivera G, Schwarz Y, Ghosh SS, Maumet C, Sochat VV, Nichols TE, Poldrack RA, Poline JB, 2015. NeuroVault.org: a web-based repository for collecting and sharing unthresholded statistical maps of the human brain. Front Neuroinformatics 9: 8. 10.3389/fninf.2015.00008 PMC4392315 25914639

[GR262113DUMC31] Gould DB, Walter MA. 2004. Mutational analysis of *BARHL1* and *BARX1* in three new patients with Joubert syndrome. Am J Med Genet A 131A: 205–208. 10.1002/ajmg.a.30227 15487006

[GR262113DUMC32] The GTEx Consortium. 2015. The Genotype-Tissue Expression (GTEx) pilot analysis: multitissue gene regulation in humans. Science 348: 648–660. 10.1126/science.1262110 25954001 PMC4547484

[GR262113DUMC33] Guo Y, Dai Y, Yu H, Zhao S, Samuels DC, Shyr Y. 2017. Improvements and impacts of GRCh38 human reference on high throughput sequencing data analysis. Genomics 109: 83–90. 10.1016/j.ygeno.2017.01.005 28131802

[GR262113DUMC34] Havrilla JM, Pedersen BS, Layer RM, Quinlan AR. 2019. A map of constrained coding regions in the human genome. Nat Genet 51: 88–95. 10.1038/s41588-018-0294-6 30531870 PMC6589356

[GR262113DUMC35] Hawrylycz MJ, Lein ES, Guillozet-Bongaarts AL, Shen EH, Ng L, Miller JA, van de Lagemaat LN, Smith KA, Ebbert A, Riley ZL, 2012. An anatomically comprehensive atlas of the adult human brain transcriptome. Nature 489: 391–399. 10.1038/nature11405 22996553 PMC4243026

[GR262113DUMC36] Hayward P. 2004. Joubert syndrome may provide clues about human evolution. Lancet Neurol 3: 574. 10.1016/S1474-4422(04)00870-1 15461986

[GR262113DUMC37] Hodge RD, Bakken TE, Miller JA, Smith KA, Barkan ER, Graybuck LT, Close JL, Long B, Johansen N, Penn O, 2019. Conserved cell types with divergent features in human versus mouse cortex. Nature 573: 61–68. 10.1038/s41586-019-1506-7 31435019 PMC6919571

[GR262113DUMC38] Hoischen A, van Bon BWM, Rodríguez-Santiago B, Gilissen C, Vissers LELM, de Vries P, Janssen I, van Lier B, Hastings R, Smithson SF, 2011. De novo nonsense mutations in *ASXL1* cause Bohring-Opitz syndrome. Nat Genet 43: 729–731. 10.1038/ng.868 21706002

[GR262113DUMC39] Huang Y, Xie C, Ye AY, Li CY, Gao G, Wei L. 2013. Recent adaptive events in human brain revealed by meta-analysis of positively selected genes. PLoS One 8: e61280. 10.1371/journal.pone.0061280 23593450 PMC3622023

[GR262113DUMC40] Jensen LJ, Kuhn M, Stark M, Chaffron S, Creevey C, Muller J, Doerks T, Julien P, Roth A, Simonovic M, 2009. STRING 8—a global view on proteins and their functional interactions in 630 organisms. Nucleic Acids Res 37: D412–D416. 10.1093/nar/gkn760 18940858 PMC2686466

[GR262113DUMC41] Kapheim KM, Pan H, Li C, Salzberg SL, Puiu D, Magoc T, Robertson HM, Hudson ME, Venkat A, Fischman BJ, 2015. Genomic signatures of evolutionary transitions from solitary to group living. Science 348: 1139–1143. 10.1126/science.aaa4788 25977371 PMC5471836

[GR262113DUMC42] Kerby DS. 2014. The simple difference formula: an approach to teaching nonparametric correlation. Compr Psychol 3: 11.IT.3.1. 10.2466/11.IT.3.1

[GR262113DUMC43] King MC, Wilson AC. 1975. Evolution at two levels in humans and chimpanzees. Science 188: 107–116. 10.1126/science.1090005 1090005

[GR262113DUMC44] Köhler S, Carmody L, Vasilevsky N, Jacobsen JOB, Danis D, Gourdine JP, Gargano M, Harris NL, Matentzoglu N, McMurry JA, 2019. Expansion of the human phenotype ontology (HPO) knowledge base and resources. Nucleic Acids Res 47: D1018–D1027. 10.1093/nar/gky1105 30476213 PMC6324074

[GR262113DUMC45] Kostka D, Hubisz MJ, Siepel A, Pollard KS. 2012. The role of GC-biased gene conversion in shaping the fastest evolving regions of the human genome. Mol Biol Evol 29: 1047–1057. 10.1093/molbev/msr279 22075116 PMC3278478

[GR262113DUMC46] Koziol LF, Budding DE, Chidekel D. 2012. From movement to thought: executive function, embodied cognition, and the cerebellum. Cerebellum 11: 505–525. 10.1007/s12311-011-0321-y 22068584

[GR262113DUMC47] Kryuchkova-Mostacci N, Robinson-Rechavi M. 2016. A benchmark of gene expression tissue-specificity metrics. Brief Bioinform 18: 205–214. 10.1093/bib/bbw008. 10.1093/bib/bbw008 PMC5444245 26891983

[GR262113DUMC48] Lein ES, Hawrylycz MJ, Ao N, Ayres M, Bensinger A, Bernard A, Boe AF, Boguski MS, Brockway KS, Byrnes EJ, 2007. Genome-wide atlas of gene expression in the adult mouse brain. Nature 445: 168–176. 10.1038/nature05453 17151600

[GR262113DUMC49] Lips ES, Cornelisse LN, Toonen RF, Min JL, Hultman CM, the International Schizophrenia Consortium; Holmans PA, O'Donovan MC, Purcell SM, Smit AB, 2012. Functional gene group analysis identifies synaptic gene groups as risk factor for schizophrenia. Mol Psychiatry 17: 996–1006. 10.1038/mp.2011.117 21931320 PMC3449234

[GR262113DUMC50] Loh KH. 2016. Proteomics: the proteomes of excitatory and inhibitory synaptic clefts. Nat Methods 13: 903. 10.1038/nmeth.4050

[GR262113DUMC51] Lou DI, McBee RM, Le UQ, Stone AC, Wilkerson GK, Demogines AM, Sawyer SL. 2014. Rapid evolution of *BRCA1* and *BRCA2* in humans and other primates. BMC Evol Biol 14: 155. 10.1186/1471-2148-14-155 25011685 PMC4106182

[GR262113DUMC52] Maere S, Heymans K, Kuiper M. 2005. BiNGO: a Cytoscape plugin to assess overrepresentation of Gene Ontology categories in Biological Networks. Bioinforma Oxf Engl 21: 3448–3449. 10.1093/bioinformatics/bti551 15972284

[GR262113DUMC53] McDonald JH, Kreitman M. 1991. Adaptive protein evolution at the *Adh* locus in *Drosophila*. Nature 351: 652–654. 10.1038/351652a0 1904993

[GR262113DUMC54] Mehmet D, Unal S, Gumruk F, Akarsu NA. 2016. A homozygous germ line nonsense mutation in *BRCA1* leading Fanconi anemia and neuroblastoma. Blood 128: 5073–5073. 10.1182/blood.V128.22.5073.5073

[GR262113DUMC55] Mekel-Bobrov N, Gilbert SL, Evans PD, Vallender EJ, Anderson JR, Hudson RR, Tishkoff SA, Lahn BT. 2005. Ongoing adaptive evolution of *ASPM*, a brain size determinant in *Homo sapiens*. Science 309: 1720–1722. 10.1126/science.1116815 16151010

[GR262113DUMC56] Merico D, Isserlin R, Stueker O, Emili A, Bader GD. 2010. Enrichment Map: a network-based method for gene-set enrichment visualization and interpretation. PLoS One 5: e13984. 10.1371/journal.pone.0013984 21085593 PMC2981572

[GR262113DUMC57] Miyata T, Kuma K, Iwabe N, Nikoh N. 1994. A possible link between molecular evolution and tissue evolution demonstrated by tissue specific genes. Jpn J Genet 69: 473–480. 10.1266/jjg.69.473 7999368

[GR262113DUMC58] Montgomery SH, Mundy NI, Barton RA. 2014. *ASPM* and mammalian brain evolution: a case study in the difficulty in making macroevolutionary inferences about gene-phenotype associations. Proc R Soc B Biol Sci 281: 20131743. 10.1098/rspb.2013.1743 PMC3906929 24452019

[GR262113DUMC59] Neubauer S, Hublin JJ, Gunz P. 2018. The evolution of modern human brain shape. Sci Adv 4: eaao5961. 10.1126/sciadv.aao5961 29376123 PMC5783678

[GR262113DUMC60] Nielsen R, Bustamante C, Clark AG, Glanowski S, Sackton TB, Hubisz MJ, Fledel-Alon A, Tanenbaum DM, Civello D, White TJ, 2005. A scan for positively selected genes in the genomes of humans and chimpanzees. PLoS Biol 3: e170. 10.1371/journal.pbio.0030170 15869325 PMC1088278

[GR262113DUMC61] Nowakowski TJ, Bhaduri A, Pollen AA, Alvarado B, Mostajo-Radji MA, Lullo ED, Haeussler M, Sandoval-Espinosa C, Liu SJ, Velmeshev D, 2017. Spatiotemporal gene expression trajectories reveal developmental hierarchies of the human cortex. Science 358: 1318–1323. 10.1126/science.aap8809 29217575 PMC5991609

[GR262113DUMC62] Nuttle X, Giannuzzi G, Duyzend MH, Schraiber JG, Narvaiza I, Sudmant PH, Penn O, Chiatante G, Malig M, Huddleston J, 2016. Emergence of a *Homo sapiens*-specific gene family and chromosome 16p11.2 CNV susceptibility. Nature 536: 205–209. 10.1038/nature19075 27487209 PMC4988886

[GR262113DUMC63] O'Toole ÁN, Hurst LD, McLysaght A. 2018. Faster evolving primate genes are more likely to duplicate. Mol Biol Evol 35: 107–118. 10.1093/molbev/msx270 29126243 PMC5850500

[GR262113DUMC64] Pao GM, Zhu Q, Perez-Garcia CG, Chou SJ, Suh H, Gage FH, O'Leary DDM, Verma IM. 2014. Role of BRCA1 in brain development. Proc Natl Acad Sci 111: E1240–E1248. 10.1073/pnas.1400783111 24639535 PMC3977248

[GR262113DUMC65] Paracchini S, Thomas A, Castro S, Lai C, Paramasivam M, Wang Y, Keating BJ, Taylor JM, Hacking DF, Scerri T, 2006. The chromosome 6p22 haplotype associated with dyslexia reduces the expression of *KIAA0319*, a novel gene involved in neuronal migration. Hum Mol Genet 15: 1659–1666. 10.1093/hmg/ddl089 16600991

[GR262113DUMC66] Paten B, Herrero J, Beal K, Fitzgerald S, Birney E. 2008. Enredo and Pecan: genome-wide mammalian consistency-based multiple alignment with paralogs. Genome Res 18: 1814–1828. 10.1101/gr.076554.108 18849524 PMC2577869

[GR262113DUMC67] Pollard KS, Salama SR, Lambert N, Lambot MA, Coppens S, Pedersen JS, Katzman S, King B, Onodera C, Siepel A, 2006. An RNA gene expressed during cortical development evolved rapidly in humans. Nature 443: 167–172. 10.1038/nature05113 16915236

[GR262113DUMC68] Pulvers JN, Huttner WB. 2009. Brca1 is required for embryonic development of the mouse cerebral cortex to normal size by preventing apoptosis of early neural progenitors. Development 136: 1859–1868. 10.1242/dev.033498 19403657

[GR262113DUMC69] Rao SM, Mayer AR, Harrington DL. 2001. The evolution of brain activation during temporal processing. Nat Neurosci 4: 317–323. 10.1038/85191 11224550

[GR262113DUMC70] Ruano D, Abecasis GR, Glaser B, Lips ES, Cornelisse LN, de Jong APH, Evans DM, Smith GD, Timpson NJ, Smit AB, 2010. Functional gene group analysis reveals a role of synaptic heterotrimeric G proteins in cognitive ability. Am J Hum Genet 86: 113–125. 10.1016/j.ajhg.2009.12.006 20060087 PMC2820181

[GR262113DUMC71] Salvador-Martínez I, Coronado-Zamora M, Castellano D, Barbadilla A, Salazar-Ciudad I. 2018. Mapping selection within *Drosophila melanogaster* embryo's anatomy. Mol Biol Evol 35: 66–79. 10.1093/molbev/msx266 29040697

[GR262113DUMC72] Sanders SJ, He X, Willsey AJ, Ercan-Sencicek AG, Samocha KE, Cicek AE, Murtha MT, Bal VH, Bishop SL, Dong S, 2015. Insights into autism spectrum disorder genomic architecture and biology from 71 risk loci. Neuron 87: 1215–1233. 10.1016/j.neuron.2015.09.016 26402605 PMC4624267

[GR262113DUMC73] Schoenemann PT, Sheehan MJ, Glotzer LD. 2005. Prefrontal white matter volume is disproportionately larger in humans than in other primates. Nat Neurosci 8: 242–252. 10.1038/nn1394 15665874

[GR262113DUMC74] Shannon P, Markiel A, Ozier O, Baliga NS, Wang JT, Ramage D, Amin N, Schwikowski B, Ideker T. 2003. Cytoscape: a software environment for integrated models of biomolecular interaction networks. Genome Res 13: 2498–2504. 10.1101/gr.1239303 14597658 PMC403769

[GR262113DUMC75] Stoletzki N, Eyre-Walker A. 2011. Estimation of the neutrality index. Mol Biol Evol 28: 63–70. 10.1093/molbev/msq249 20837603

[GR262113DUMC76] Su AI, Wiltshire T, Batalov S, Lapp H, Ching KA, Block D, Zhang J, Soden R, Hayakawa M, Kreiman G, 2004. A gene atlas of the mouse and human protein-encoding transcriptomes. Proc Natl Acad Sci 101: 6062–6067. 10.1073/pnas.0400782101 15075390 PMC395923

[GR262113DUMC77] Sundaram L, Gao H, Padigepati SR, McRae JF, Li Y, Kosmicki JA, Fritzilas N, Hakenberg J, Dutta A, Shon J, 2018. Predicting the clinical impact of human mutation with deep neural networks. Nat Genet 50: 1161–1170. 10.1038/s41588-018-0167-z 30038395 PMC6237276

[GR262113DUMC78] Suzuki IK, Gacquer D, Van Heurck R, Kumar D, Wojno M, Bilheu A, Herpoel A, Lambert N, Cheron J, Polleux F, 2018. Human-specific *NOTCH2NL* genes expand cortical neurogenesis through Delta/Notch regulation. Cell 173: 1370–1384.e16. 10.1016/j.cell.2018.03.067 29856955 PMC6092419

[GR262113DUMC79] Tao X, West AE, Chen WG, Corfas G, Greenberg ME. 2002. A calcium-responsive transcription factor, CaRF, that regulates neuronal activity-dependent expression of BDNF. Neuron 33: 383–395. 10.1016/S0896-6273(01)00561-X 11832226

[GR262113DUMC80] Tasic B, Yao Z, Graybuck LT, Smith KA, Nguyen TN, Bertagnolli D, Goldy J, Garren E, Economo MN, Viswanathan S, 2018. Shared and distinct transcriptomic cell types across neocortical areas. Nature 563: 72–78. 10.1038/s41586-018-0654-5 30382198 PMC6456269

[GR262113DUMC81] Tuller T, Kupiec M, Ruppin E. 2008. Evolutionary rate and gene expression across different brain regions. Genome Biol 9: R142. 10.1186/gb-2008-9-9-r142 18811952 PMC2592720

[GR262113DUMC82] Unger S, Górna MW, Le Béchec A, Do Vale-Pereira S, Bedeschi MF, Geiberger S, Grigelioniene G, Horemuzova E, Lalatta F, Lausch E, 2013. *FAM111A* mutations result in hypoparathyroidism and impaired skeletal development. Am J Hum Genet 92: 990–995. 10.1016/j.ajhg.2013.04.020 23684011 PMC3675238

[GR262113DUMC83] Varki A, Geschwind DH, Eichler EE. 2008. Human uniqueness: genome interactions with environment, behaviour and culture. Nat Rev Genet 9: 749–763. 10.1038/nrg2428 18802414 PMC2756412

[GR262113DUMC84] Wang HY, Chien HC, Osada N, Hashimoto K, Sugano S, Gojobori T, Chou CK, Tsai SF, Wu CI, Shen CKJ. 2006. Rate of evolution in brain-expressed genes in humans and other primates. PLoS Biol 5: e13. 10.1371/journal.pbio.0050013 PMC1717015 17194215

[GR262113DUMC85] Wang K, Li M, Hakonarson H. 2010. ANNOVAR: functional annotation of genetic variants from high-throughput sequencing data. Nucleic Acids Res 38: e164. 10.1093/nar/gkq603 20601685 PMC2938201

[GR262113DUMC86] Whitney O, Pfenning AR, Howard JT, Blatti CA, Liu F, Ward JM, Wang R, Audet JN, Kellis M, Mukherjee S, 2014. Core and region-enriched networks of behaviorally regulated genes and the singing genome. Science 346: 1256780. 10.1126/science.1256780 25504732 PMC4359888

[GR262113DUMC87] Xu X, Wells AB, O'Brien DR, Nehorai A, Dougherty JD. 2014. Cell type-specific expression analysis to identify putative cellular mechanisms for neurogenetic disorders. J Neurosci 34: 1420–1431. 10.1523/JNEUROSCI.4488-13.2014 24453331 PMC3898298

[GR262113DUMC88] Yamamoto M, McCaffery P, Dräger UC. 1996. Influence of the choroid plexus on cerebellar development: analysis of retinoic acid synthesis. Dev Brain Res 93: 182–190. 10.1016/0165-3806(96)00038-7 8804705

[GR262113DUMC89] Yang Z. 2007. PAML 4: phylogenetic analysis by maximum likelihood. Mol Biol Evol 24: 1586–1591. 10.1093/molbev/msm088 17483113

[GR262113DUMC90] Yang Z, Bielawski J. 2000. Statistical methods for detecting molecular adaptation. Trends Ecol Evol 15: 496–503. 10.1016/S0169-5347(00)01994-7 11114436 PMC7134603

[GR262113DUMC91] Yu F, Hill RS, Schaffner SF, Sabeti PC, Wang ET, Mignault AA, Ferland RJ, Moyzis RK, Walsh CA, Reich D. 2007. Comment on “Ongoing adaptive evolution of *ASPM*, a brain size determinant in *Homo sapiens*”. Science 316: 370. 10.1126/science.1137568 17446375

[GR262113DUMC92] Zylka MJ, Simon JM, Philpot BD. 2015. Gene length matters in neurons. Neuron 86: 353–355. 10.1016/j.neuron.2015.03.059 25905808 PMC4584405

