# Supplementary material for: Systematic detection of brain protein-coding genes under positive selection during primate evolution and their roles in cognition
Source: Genome Res. 2021 Mar;31(3):484–96. doi: 10.1101/gr.262113.120 (PMC7919455; doi:10.1101/gr.262113.120)
Supplement: Supplemental Material [file supp_31_3_484__index.html]

Systematic detection of brain protein-coding genes under positive selection during primate evolution and their roles in cognition — Supplemental Material 

# Systematic detection of brain protein-coding genes under positive selection during primate evolution and their roles in cognition

## Supplemental Material

- Supplemental\_Methods\_and\_Figures.pdf
- Supplemental\_Tables.zip
- Supplemental\_Material.zip
