# Supplementary material for: Systematic detection of brain protein-coding genes under positive selection during primate evolution and their roles in cognition
Source: Genome Res. 2021 Mar;31(3):484–96. doi: 10.1101/gr.262113.120 (PMC7919455; doi:10.1101/gr.262113.120)
Supplement: Supplemental Material [file supp_gr.262113.120_Supplemental_Material.zip › src/public/app/components/adult-human-brain/adult-human-brain.html]

Adult human brain

Documentation

{{ map.legends.R1 }}

{{ map.legends.R2 }}


{{ map.legends.R3 }}


{{ map.legends.R4 }}

{{ map.legends.R4 }}

Reverse specificity
High specificity
