# Supplementary material for: Systematic detection of brain protein-coding genes under positive selection during primate evolution and their roles in cognition
Source: Genome Res. 2021 Mar;31(3):484–96. doi: 10.1101/gr.262113.120 (PMC7919455; doi:10.1101/gr.262113.120)
Supplement: Supplemental Material [file supp_gr.262113.120_Supplemental_Material.zip › src/public/app/components/bootstrap/bootstrap.html]

Bootstrap

Documentation

## Evolution of your gene set vs genome

### Calculate with bootstrap on human dN/dS

More divergent
Non-significant
More conserved
p-value < 0.05
p-value > 0.05

**Gene set median**
{{ median }}

**Bootstrap confidence interval**
[
{{ confidenceInterval.low }}
{{ confidenceInterval.high }}
]
