# Supplementary material for: Systematic detection of brain protein-coding genes under positive selection during primate evolution and their roles in cognition
Source: Genome Res. 2021 Mar;31(3):484–96. doi: 10.1101/gr.262113.120 (PMC7919455; doi:10.1101/gr.262113.120)
Supplement: Supplemental Material [file supp_gr.262113.120_Supplemental_Material.zip › src/public/app/components/human-hemisphere/human-hemisphere.html]

Human left hemisphere

Documentation

Displays the median of the expression levels or specificity in the brain for the selected gene(s).

Click on the brain to highlight a particular region or select it in the menu below.
Double click outside the brain to reset the view.

Currently displayed:
**median of all genes.**
**only {{ brainGene.gene.Gene }}.**

Reset

Specificity
Expression

Low expression
Average expression
High expression

Reverse specificity
No specificity
High specificity
