# Supplementary material for: Systematic detection of brain protein-coding genes under positive selection during primate evolution and their roles in cognition
Source: Genome Res. 2021 Mar;31(3):484–96. doi: 10.1101/gr.262113.120 (PMC7919455; doi:10.1101/gr.262113.120)
Supplement: Supplemental Material [file supp_gr.262113.120_Supplemental_Material.zip › src/public/app/components/search/search.html]

GenEvo is an online tool that enables users to explore, visualize, and test the evolution of protein-coding genes in modern and archaic humans, and other primates.

Users can explore their own gene list or curated gene list. Once queried, GenEvo computes the direction and magnitude of selective pressure of the gene list compared to the genome. It also proposes different visualizations, from raw evolutionary measure (dN/dS; Neanderthal introgression) to brain expression (3D visualization of Allen Brain atlas).

All outputs can be downloaded in TSV format for further analyses.


Search

Reset

Download
