# Supplementary material for: Systematic detection of brain protein-coding genes under positive selection during primate evolution and their roles in cognition
Source: Genome Res. 2021 Mar;31(3):484–96. doi: 10.1101/gr.262113.120 (PMC7919455; doi:10.1101/gr.262113.120)
Supplement: Supplemental Material [file supp_gr.262113.120_Supplemental_Material.zip › src/public/app/components/sidebar/sidebar.html]

No genes to display

Genes found
{{ genes.length }}

Genes selected
{{ selectedDots.length }}

Copy to clipboard

{{ currentPage }} / {{ totalPages || 1 }}

- {{ gene.Gene }}

  - Show variants
  - Brain specificity
  - Brain expression
  - Download DNA
  - Download protein
  - See on NCBI
  - See on Ensembl
  - See on BioGPS
  - See on GTEx
  - See on gene2pheno
  - See on DBD
  - See on OMIM
