# Supplementary figures and images for: Systematic detection of brain protein-coding genes under positive selection during primate evolution and their roles in cognition
Source: Genome Res. 2021 Mar;31(3):484–96. doi: 10.1101/gr.262113.120 (PMC7919455; doi:10.1101/gr.262113.120)

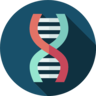

Supplement: Supplemental Material [file supp_gr.262113.120_Supplemental_Material.zip › src/public/app/images/favicon.png]

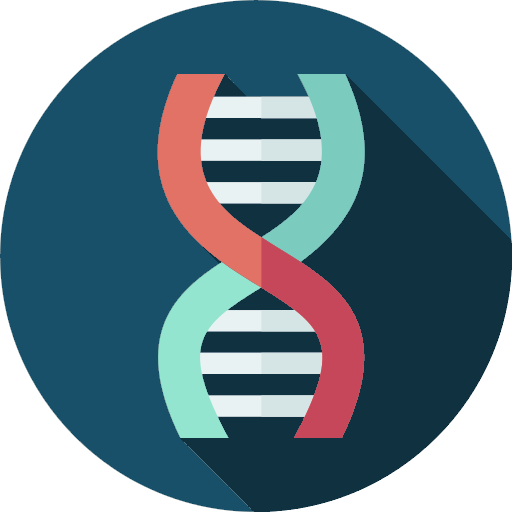

Supplement: Supplemental Material [file supp_gr.262113.120_Supplemental_Material.zip › src/public/app/images/logo-genevo.png]

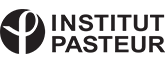

Supplement: Supplemental Material [file supp_gr.262113.120_Supplemental_Material.zip › src/public/app/images/logo-pasteur.png]

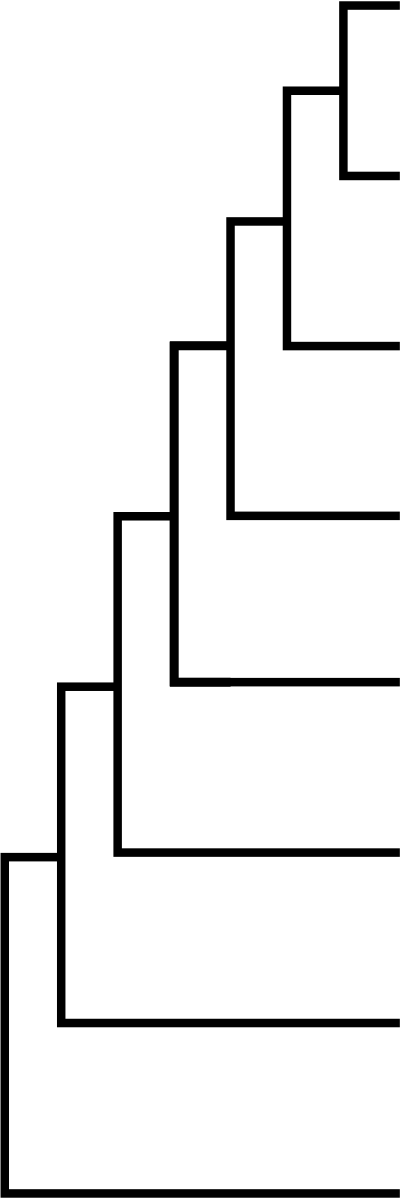

Supplement: Supplemental Material [file supp_gr.262113.120_Supplemental_Material.zip › src/public/app/images/phylotree.png]
